# Draft Genome Sequence of a Clinical Isolate of *Mycobacterium tuberculosis* Strain PR05

**DOI:** 10.1128/genomeA.00397-13

**Published:** 2013-06-20

**Authors:** Azwin Ismail, L. K. Teh, Y. F. Ngeow, M. N. Norazmi, Zainuddin F. Zainul, T. H. Tang, N. Najimudin, M. Z. Salleh

**Affiliations:** Pharmacogenomics Centre (PROMISE), Faculty of Pharmacy, Universiti Teknologi MARA Malaysia, Puncak Alam Campus, Selangor, Malaysiaa; Faculty of Medicine, University of Malaya, Kuala Lumpur, Malaysiab; School of Health Sciences, Health Campus, Universiti Sains Malaysia, Kubang Kerian, Kelantan, Malaysiac; Institute for Research in Molecular Medicine (INFORMM), Universiti Sains Malaysia, Pulau Pinang, Malaysiad; Advanced Medical and Dental Institute, Universiti Sains Malaysia, Kepala Batas, Pulau Pinang, Malaysiae; School of Biological Sciences, Universiti Sains Malaysia, Pulau Pinang, Malaysiaf

## Abstract

We report the annotated genome sequence of a clinical isolate, *Mycobacterium tuberculosis* strain PR05, which was isolated from the human cerebrospinal fluid of a patient diagnosed with tuberculosis.

## GENOME ANNOUNCEMENT

Tuberculosis meningitis (TM) is a severe manifestation of extrapulmonary tuberculosis in humans caused by *Mycobacterium tuberculosis*. It is a slow progressive granulomatous inflammation of the basal meninges that can lead to various complications such as cerebral vascular infarction, hydrocephalus, and cranial nerve palsy (1). TM is also known to cause fatal tuberculosis disease associated with a high frequency of neurologic sequelae and mortality (2–6).

Here we report a draft genome sequence of a clinical isolate of *Mycobacterium tuberculosis*, strain PR05, isolated from the human cerebrospinal fluid of a patient diagnosed with tuberculosis. The whole-genome sequencing of *Mycobacterium tuberculosis* strain PR05 was performed using an Illumina Genome Analyzer IIx sequencer with sequencing by synthesis (SBS) technology (7). A total of 25,189,322 nucleotides were generated and 62,307 reads were trimmed off by 99.75%, providing 205.13-fold genome coverage and four megabases of genome size. These reads were assembled using CLCbio (CLC Genomics Workbench version 5.5.1). OSlay was used for ordering and sorting out the assemblies of *M. tuberculosis* strain PR05 (8). The draft genome was annotated using the automated annotation server RAST (Rapid Annotation using Subsystem Technology) (9) and BASys (Bacterial Annotation System) (10).

In *de novo* assembly, the generated 25,065,512 reads covered a total of 887,991,375 bp. The genome of PR05 has a G+C content of 65.6% with approximately 1,412,995 read counts. The number of contigs derived from the *de novo* assembly was 225, ranging from 200 to 181,900 bp with an average length of 19,243 bp and an N_50_ of 64,609 bp. The number of contigs greater than 500 bp was 180. Strain PR05 was mapped to H37Rv (11) and the CCDC5180 reference strain (12). The PR05 genome is a circular DNA of 4,410,213 bp, which is 1,319 bp shorter than the virulent H37Rv strain and 4,232 bp longer than the Beijing CCDC5180 strain. The genome was most similar to strain H37Rv, with 99% similarity in the nucleotide sequence over the alignable portions by using fast sequence alignment tools BLAST (13).

The annotation for the PR05 genome sequence comprises 4,184 coding sequences and 44 RNA genes. We found 394 subsystems, compared to 368 subsystems found in the H37Rv strain. We detected 109 protein-encoding genes, which may play a role in virulence and invasion. Another 86 protein-encoding genes that are related to invasion and intracellular resistance and 22 protein-encoding genes that are involved in antibiotic resistance were detected. A total of 4,771 genes were identified and 4,768 genes were annotated through the BASys.

### Nucleotide sequence accession numbers.

This whole-genome shotgun project has been deposited at DDBJ/EMBL/GenBank under the accession number AOMG00000000. The version described in this paper is the second version, AOMG02000000.
